# Evaluation of the Antimicrobial and Anti-inflammatory Properties of *Bacillus*-DFM (Norum™) in Broiler Chickens Infected With *Salmonella* Enteritidis

**DOI:** 10.3389/fvets.2019.00282

**Published:** 2019-08-27

**Authors:** Bishnu Adhikari, Daniel Hernandez-Patlan, Bruno Solis-Cruz, Young Min Kwon, Margarita A. Arreguin, Juan D. Latorre, Xochitl Hernandez-Velasco, Billy M. Hargis, Guillermo Tellez-Isaias

**Affiliations:** ^1^Department of Poultry Science, University of Arkansas, Fayetteville, AR, United States; ^2^Unidad de Investigación Multidisciplinaria, Laboratorio 5: LEDEFAR, Facultad de Estudios Superiores (FES) Cuautitlán, Universidad Nacional Autónoma de México (UNAM), Cuautitlán Izcalli, Mexico; ^3^Eco-Bio LLC, Fayetteville, AR, United States; ^4^Departamento de Medicina y Zootecnia de Aves, Facultad de Medicina Veterinaria y Zootecnia, Universidad Nacional Autónoma de México, Mexico City, Mexico

**Keywords:** *Bacillus*, broiler chickens, *Salmonella* Enteritidis, antimicrobial, anti-inflammatory (activity)

## Abstract

Restrictions of in-feed antibiotics use in poultry has pushed research toward finding appropriate alternatives such as Direct-Fed Microbials (DFM). In this study, previously tested *Bacillus* isolates (*B*. *subtilis* and *B*. *amyloliquefaciens*) were used to evaluate their therapeutic and prophylactic effects against *Salmonella enterica* serovar Enteritidis (*S*. Enteritidis) in broiler chickens. For this purpose, initial antibacterial activity of *Bacillus*-DFM (10^4^ spores/g or 10^6^ spores/g) against *S*. Enteritidis colonization in crop, proventriculus and intestine was investigated using an *in vitro* digestive model. Furthermore, to evaluate therapeutic and prophylactic effects of *Bacillus*-DFM (10^4^ spores/g) against *S*. Enteritidis colonization, altogether 60 (*n* = 30/group) and 30 (*n* = 15/group) 1-day-old broiler chickens were randomly allocated to either DFM or control group (without *Bacillus*-DFM), respectively. Chickens were orally gavaged with 10^4^ cfu of *S*. Enteritidis per chicken at 1-day old, and cecal tonsils (CT) and crop were collected 3 and 10 days later during the therapeutic study, whereas they were orally gavaged with 10^7^ cfu of *S*. Enteritidis per chicken at 6-day-old, and CT and crop were collected 24 h later from two independent trials during the prophylactic study. Serum superoxide dismutase (SOD), FITC-d and intestinal IgA levels were reported for both chicken studies, in addition cecal microbiota analysis was performed during the therapeutic study. DFM significantly reduced *S*. Enteritidis concentration in the intestine compartment, and in both proventriculus and intestine compartments as compared to the control when used at 10^4^ spores/g and 10^6^ spores/g, respectively (*p* < 0.05). DFM significantly reduced FITC-d and IgA as well as SOD and IgA levels (*p* < 0.05) compared to the control in therapeutic and prophylactic studies, respectively. Interestingly, in the therapeutic study, there were significant differences in bacterial community structure and predicted metabolic pathways between DFM and control. Likewise, phylum *Actinobacteria* and the genera *Bifidobacterium, Roseburia, Proteus*, and cc_115 were decreased, while the genus *Streptococcus* was enriched significantly in the DFM group as compared to the control (MetagenomeSeq, *p* < 0.05). Thus, the overall results suggest that the *Bacillus*-DFM can reduce *S*. Enteritidis colonization and improve the intestinal health in chickens through mechanism(s) that might involve the modulation of gut microbiota and their metabolic pathways.

## Introduction

Antibiotics have been widely used in animal production for decades not only for therapeutic purposes, but also as antimicrobial growth promoters (AGPs) to enhance growth rate and feed conversion efficiency (1, 2). Although the use of AGPs has a significant positive economic impact in commercial animal production systems, there is a greater concern regarding possibilities of their use in developing antimicrobial resistance (AMR) in bacterial populations. Because of this reason, the use of in-feed antibiotics has been completely banned in Europe since January 1st, 2006 (EC Regulation No. 1831/2003) and has also been restricted in several non-European countries, including Taiwan and South Korea (3). Since January 2017, medically important antibiotics to human health are no longer allowed in animal production for growth promotion or feed efficiency in the United States and require licensed veterinarian prescription to use them for prevention, control, and treatment of animal diseases (FDA's Guidance #213).

The poultry industry is the fastest growing animal industry and is expected to grow continuously as demand for meat and eggs is accelerating due to growing populations, increasing incomes and urbanization (4). However, due to ban or restrictions on AGPs, there are growing challenges for the poultry industry to cope with enteric pathogens such as *Salmonella*. This has created huge demand for finding alternatives to AGPs, and thus several possible alternatives such as enzymes (in), organic acids, probiotics, prebiotics, etheric oils, and immunostimulants have already been widely studied (2, 5).

Among those alternatives, probiotics or Direct-Fed Microbials (DFM), which were defined as “a live microbial feed supplement that beneficially affects the host animal by improving its intestinal microbial balance” (6), have generated significant interest during the last two decades to all sectors of animal production. The majority of microbes used as DFM are bacteria that belong to around 40 different species in 7 bacterial genera including *Lactobacillus, Bifidobacterium, Propionbacterium, Enterococcus, Pediococcus, Bacillus*, and *Bacteroides*. In addition to these bacteria, yeast (*Saccharomyces cerevisiae*) and molds (*Aspergillus niger* and *Aspergillus oryzae*) were also reported as DFM (7). Moreover, certain strains of *Clostridium* such as *Clostridium butyricum* MIYAIRI 588 were also used as potential probiotics (8). Unlike other bacteria whose vegetative cells are used as DFM, spores from *Bacillus* sps. can be used as DFM because they are more stable and heat tolerant (9–11), and thus well-suited for its application in pelleted feeds (12). Previous studies reported the ability of *Bacillus* spores to germinate and enumerate within the gastrointestinal tract of the poultry (13–15). In poultry, several studies have reported beneficial effects of *Bacillus* isolates when used as DFM on production parameters and pathogen inhibition (16–18), which might be achieved through increasing nutrient digestibility, improving intestinal morphology, balancing intestinal microbiota, and modulating immunity (19–21). Moreover, our previous studies based on the selected candidates of *Bacillus* sps. reported the reduction in the recovery of *Salmonella* Typhimurium in both chicks and poults after experimental infection in preliminary laboratory trials (22) as well as in poults during the brooding phase of commercial turkey production (12). However, the modes of action for improved performance by *Bacillus* species were not well-defined, and performance parameters were varied within species or strains, demanding appropriate screening and characterization of *Bacillus* isolates prior to commercialization (23).

Norum™ (Eco-Bio/Euxxis Bioscience LLC, Fayetteville, AR) is a *Bacillus* spore direct DFM culture consisting of two isolates of *Bacillus amyloliquefaciens* and one isolate of *Bacillu subtilis* which were isolated in our laboratory and screened based on *in vitro* enzyme production profiles and *Clostridium perfringens* reduction (24). In addition, these isolates were shown to reduce digesta viscoscity, bacterial translocation, improve performance, bone quality parameters, and balance intestinal microbiota in chickens raised with rye-based diets or corn distiller-dried grains with solubles (21, 25). However, the effect of dietary supplementation of Norum™ has not been evaluated *in vivo* in an established *Salmonella* challenge model until now. Thus, the objectives of this study were to evaluate the antimicrobial effects of Norum™ DFM against *S*. Enteritidis in an *in vitro* digestion model that simulates the pH and enzymatic conditions present in the crop, proventriculus, and intestine of broiler chickens, as well as the therapeutic and prophylactic effects against *S*. Enteritidis colonization in crop and cecal tonsil (CT), aside from its effects on intestinal health parameters, and cecal microbiota composition in broiler chickens.

## Materials and Methods

### Preparation of Treatments and Diets

Norum™ (Eco-Bio/Euxxis Bioscience LLC, Fayetteville, AR) is a *Bacillus* spore DFM culture consisting of three isolates: two *Ba*c*illus amyloliquefaciens* and one *Bacillu subtilis*. The product contains a concentration of stable *Bacillus* spores (~3 × 10^11^ spores/g). DFM was added into the feed to obtain the experimental diet with a final concentration of 10^4^ or 10^6^ spores/g feed. Samples of feed containing the DFM were subjected to 100°C for 10 min to eliminate vegetative cells and validate the number of spores per gram of feed after inclusion and mixing steps. Following heat treatment, 10-fold dilutions of the feed samples were plated on TSA, letting spores in the feed sample germinate to vegetative cells after incubation at 37°C for 24 h, hence representing the number of spores present per gram of feed. The experimental diet used in this study was formulated to approximate the nutritional requirements of broiler chickens as recommended by the National Research Council (26), and adjusted to breeder's recommendations (27). No antibiotics were added to the diet (Supplementary Table 1). All animal handling procedures complied with the Institutional Animal Care and Use Committee (IACUC) at the University of Arkansas, Fayetteville (protocol #18030).

### Bacterial Strain and Culture Conditions

The organism used in all experiments was a poultry isolate of *Salmonella enterica* serovar Enteritidis (*S*. Enteritidis), bacteriophage type 13A, obtained from the USDA National Veterinary Services Laboratory (Ames, IA, United State). This strain was resistant to 25 μg/mL of novobiocin (NO, catalog no.N-1628, Sigma) and was selected for resistance to 20 μg/mL of nalidixic acid (NA, catalog no.N-4382, Sigma) in our laboratory. For the present studies, 100 μL of *S*. Enteritidis from a frozen aliquot was added to 10 mL of tryptic soy broth (Catalog no. 22092, Sigma), incubated at 37°C for 8 h, and passed three times every 8 h to ensure that all bacteria were in log phase as previously described (28). Post-incubation, bacterial cells were washed three times with sterile 0.9% saline by centrifugation at 1,864 × g for 10 min, reconstituted in saline, quantified by densitometry with a spectrophotometer (Spectronic 20D+, Spectronic Instruments Thermo Scientific, Rochester, NY, United States), and finally diluted to an approximate concentration of 1 × 10^8^, 4 × 10^4^, and 4 × 10^7^ cfu/mL. Concentrations of *S*. Enteritidis were further verified by serial dilution and plating on brilliant green agar (BGA, Catalog no. 70134, Sigma) with NO and NA for enumeration of actual cfu used to in the experiments.

### Experiment 1. *In vitro* Digestion Model

In this experiment, the antimicrobial activity of two different concentrations of DFM (10^4^ or 10^6^ spores/g) against *S*. Enteritidis was determined using an *in vitro* digestion model described previously (24, 29) that simulates the pH and enzymatic conditions present in the crop, proventriculus, and intestine of broilers. Experiments were run in quintuplicate. Briefly, 5 g of feed with or without DFM was placed inside 50 mL polypropylene centrifuge tubes, followed by the addition of 1 ml of 1 × 10^8^ cfu/mL *S*. Enteritidis suspension in each tube. Subsequently, the media and corresponding enzymes to simulate each compartment of the *in vitro* digestion model were added to the tubes, respecting the stirring conditions and incubation times established. Finally, in each compartment, 1 mL of sample was collected to enumerate *S*. Enteritidis.

### Experiment 2. Effect of Therapeutic Administration of DFM on *S*. Enteritidis

This experiment was performed to evaluate the therapeutic effect of 10^4^ spores/g DFM in broiler chickens infected with *S*. Enteritidis. Sixty 1 day-old male Cobb-Vantress broiler chickens (Fayetteville, AR, USA) were challenged with 1 × 10^4^ *S*. Enteritidis cfu per bird and randomly allocated to one of two groups (*n* = 30 chickens/group): (1) control group challenged only with *S*. Enteritidis and (2) DFM group challenged with *S*. Enteritidis and also with 10^4^ spores/g Norum™. On days 3 and 10 post-*S*. Enteritidis challenge, 15 chickens were euthanized by CO_2_ inhalation, and the crop and CT from 12 birds per group were aseptically collected to evaluate *S*. Enteritidis recovery. Blood samples were collected from the femoral vein and centrifuged (1,000 × g for 15 min) to separate the serum for the determination of fluorescein isothiocyanate-dextran (FITC-d) concentration and superoxide dismutase (SOD) activity at day 10. The concentration of FITC-d administered was calculated based on group body weight at day 9 post-*S*. Enteritidis challenge. Furthermore, intestinal samples for total intestinal IgA levels were also collected.

### Experiment 3. Effect of Prophylactic Administration of *DFM* on *S*. Enteritidis

In this experiment, two independent trials were conducted to evaluate the prophylactic administration of 10^4^ spores/g DFM in reducing the incidence of *S*. Enteritidis in broiler chickens. In each trial, 30 day-of-hatch male Cobb-Vantress broiler chickens (Fayetteville, AR, USA) were randomly allocated to one of two groups (*n* = 15 chickens): (1) control group challenged only with *S*. Enteritidis and (2) DFM group challenged with *S*. enteritidis and also with 10^4^ spores/g Norum™. Chicks were placed in heated brooder batteries with a controlled age-appropriate environment and provided with their respective diet and water *ad libitum*. At day 6, all chickens were orally gavaged with 1 × 10^7^ cfu of *S*. Enteritidis per bird. Chicks were euthanized by CO_2_ inhalation 24 h post-*S*. Enteritidis challenge, and the crop and CT from 12 birds per group were aseptically collected to evaluate *S*. Enteritidis recovery. Blood samples were collected from the femoral vein and centrifuged (1,000 × g for 15 min) to separate the serum for the determination of FITC-d and SOD. The concentration of FITC-d administered was calculated based on group body weight at 6 d old. Furthermore, intestinal samples for total intestinal IgA levels were also collected.

### *Salmonella* Recovery

The crop and CT collected in experiments 2 and 3 were homogenized and diluted with saline (1:4 w/v), and 10-fold dilutions were plated on BGA with NO and NA, incubated at 37°C for 24 h to enumerate total *S*. Enteritidis colony forming units. Following plating to enumerate total *S*. Enteritidis, the crop and CT samples were enriched in double strength tetrathionate enrichment broth and further incubated at 37°C for 24 h. Enrichment samples were streaked onto Xylose Lysine Tergitol-4 (XLT-4, Catalog No. 223410, BD DifcoTM) selective media for confirmation of *Salmonella* presence.

### Serum Determination of FITC-d Leakage

FITC-d (MW 3-5 KDa; Sigma-Aldrich Co., St. Louis, MO) was used as a marker of paracellular transport and mucosal barrier dysfunction (30, 31). In both *in vivo* experiments, 1 h before the chicks were euthanized by CO_2_ inhalation, 12 broiler chickens from each group were given an oral gavage dose of FITC-d (8.32 mg/kg of body weight) and the rest were used as controls. The concentrations of FITC-d from diluted sera (1:5 PBS) were measured fluorometrically at an excitation wavelength of 485 nm and an emission wavelength of 528 nm (Synergy HT, Multi-mode microplate reader, BioTek Instruments, Inc., VT, USA). FITC-d concentrations were reported as ng of FITC-d/mL of serum (31).

### Enzyme-Linked Immunosorbent Assay for Total IgA Levels

Total IgA levels in both *in vivo* experiments were determined in 12 gut rinse samples each as previously described (32). A commercial indirect ELISA set was used to quantify IgA according to the manufacturer's instructions (Catalog No. E30-103, Bethyl Laboratories Inc., Montgomery, TX 77356). Ninety six-well plates (Catalog No. 439454, Nunc MaxiSorp, Thermo Fisher Scientific, Rochester, NY) were used, and samples diluted to 1:100 were measured at 450 nm using an ELISA plate reader (Synergy HT, multi-mode microplate reader, BioTek Instruments, Inc., Winooski, VT, USA). Total intestinal IgA levels obtained were multiplied by the dilution factor (100) to determine the amount of chicken IgA in the undiluted samples.

### SOD Determination

SOD activity was measured in 12 serum samples per group using a commercial assay kit (item No. 706002, Cayman chemical company, Ann Arbor, Michigan, United States) following the manufacturer's instructions. The three types of SOD (Cu/Zn, Mn, and FeSOD) were determined in samples diluted to 1:5. Samples were measured at 450 nm using an ELISA plate reader (Synergy HT, multi-mode microplate reader, BioTek Instruments, Inc., Winooski, VT, USA).

### Data and Statistical Analysis

Log cfu/g of *S*. Enteritidis, total intestinal IgA, SOD activity and serum FITC-d concentrations were subjected to analysis of variance (ANOVA) as a completely randomized design using the General Linear Models procedure of SAS (33). Significant differences among the means were determined by Duncan's multiple-range test at *p* < 0.05. Enrichment data were expressed as positive/total chickens (%), and the percent recovery of *S*. Enteritidis was compared using the Chi-Squared test of independence (34), testing all possible combinations to determine the significance (*p* < 0.05).

### Cecal Microbiota Analysis

#### DNA Extraction and PCR

Six cecal samples from each group (control and DFM groups) from the therapeutic study at day 10 post-*S*. enteritidis challenge were used for the cecal microbiota study. DNA extraction, PCR, and library preparation were similar as described earlier (5, 35). In brief, about 200 mg of ileal content from each sample was used for genomic DNA extraction using QIAamp® fast DNA stool mini kit (Qiagen, Catalog # 51604) following manufacturer's instructions with addition incorporation of bead beating step. For bead beating, a pellet from each sample was resuspended in 1 ml inhibit Ex buffer provided with kit and transferred to 2 ml microcentrifuge tubes with screw cap (Thermofisher Scientific, Catalog # 3468) containing 0.25 ml of sterile 0.1 mm glass leads (BioSpec, Mfr # 11079101). Bead beating was performed using Bead mill 24 (Fisher Scientific) for 6 cycles where each cycle contained a run time of 0.30 s and stopping time of 0.11 s between each cycle. The V1-V3 region of 16S rRNA gene from each 10 ng genomic DNA samples was amplified by using unique barcoded universal primers as described previously (36). PCR was performed using Q5® High-Fidelity DNA Polymerase (NEB; New England Biolabs) in a final volume of 50 μl following manufacturer's instructions. The PCR condition included initial denaturation at 98 °C for 30 s followed by 30 cycles of exponential amplifications using denaturation at 98°C for 10 s, annealing at 58°C for 30 s, extension at 72°C for 30 s, and final extension at 72°C for 2 min. Amplicons were purified from 0.7% agarose gel, concentration was measured using a Qubit dsDNA broad range assay kit (Life Technologies, United States), and equal concentrations (20 ng/μl) of amplicons were pooled together. The purified pooled amplicons were sequenced using MiSeq Illumina 300 cycle paired end options at the University of California, Riverside (Riverside, CA, United States).

#### 16S rRNA Gene Sequence Analysis

Raw sequence reads were analyzed using Quantitative Insights into Microbial Ecology, QIIME version 1.9.1 (37) at Jetstream cloud computing platform (38, 39) using the pipelines as described previously (5, 35). Paired end reads were joined together using join_paired_ends.py command of QIIME with fastq-join option (40). After joining, barcode positions were formatted using a customized Perl script, and barcodes were removed using extract_barcodes.py command of QIIME. Split_libraries_fastq.py command of QIIME was used for demultiplexing and quality filtering of joined reads. Reads having a Phred quality score <20 were discarded. The chimeric sequences were identified using USEARCH version 6.1.544 (41), and chimeric sequences along with shorter sequences (<100 bp) were excluded for downstream analysis. The OTU picking was performed using pick_open_reference_otus.py command of QIIME with uclust method (41). Taxonomy was assigned based on green genes taxonomy and reference database version 13_8 (42) with RDP classifier (43). For further statistical analysis and visual exploration, an OTU table with taxa in plain format and a metadata file were uploaded to the MicrobiomeAnalyst tool (44). Data were filtered using the following options: minimum count 4 and low count filter based on 20% prevalence in samples. Alpha diversity analysis was calculated based on Shannon Index. Data were normalized using cumulative sum scaling before any statistical comparisons (45). Significant differences in alpha diversity among different groups were calculated based on ANOVA/*T*-test where a significant difference level was set at *p* < 0.05. Beta diversity was calculated based on Weighted UniFrac distance metric (46) and statistical comparisons among groups were performed with Analysis of Similarities method (ANOSIM). To determine differentially abundant phyla and genera among different groups, a MetagenomeSeq (45) that uses zero-inflated Gaussian fit model was used, where the level of significance was set at *p* < 0.05. PICRUSt ver. 1.1.3 (47) was further utilized to predict the functional pathways from 16S rRNA gene sequencing data using a closed OTU table created with the Greengenes database 13.8. The statistical analysis and visualization in the third level KEGG pathways predicted by PICRUSt between two groups were performed using the Statistical Analysis of Metagenomic Profiles (STAMP ver. 2.1.3) (48).

## Results

### *In vitro* Digestive Model

The antibacterial effect of DFM at two different concentrations **(**10^4^ spores/g and 10^6^ spores/g) against *S*. Enteritidis colonization in crop, proventriculus, and intestine using an *in vitro* digestive model is shown in Table 1. When DFM was used at 10^4^ spores/g of feed *S*. Enteritidis colonization in the intestinal compartment was significantly reduced (*p* < 0.05), while at a higher concentration (10^6^ spores/g) *S*. Enteritidis colonization in both proventriculus and intestinal compartments was significantly reduced (*p*<*0.05*) as compared to the control group (Table 1). However, the antibacterial effect of DFM was more pronounced at higher dose and especially in the intestinal compartment, where it reduced the *S*. Enteritidis colonization by more than 7 log_10_ and brought it to an undetectable level.

**Table 1:** Evaluation of the antibacterial activity of different DFM ratios on *S*. Enteritidis^†^ in an *in vitro* digestive model using the plating method^‡^.

**Treatment**	**Crop**	**Proventriculus**	**Intestine**
Control	7.78 ± 0.00	5.03 ± 0.12	7.23 ± 0.00
DFM (10^4^ spores/g)	7.78 ± 0.00	5.11 ± 0.03	5.31 ± 0.10
DFM (10^6^ spores/g)	7.66 ± 0.01	4.22 ± 0.04	0.00 ± 0.00

*Values within treatment columns for each treatment with different superscripts differ significantly (p < 0.05)*.

*Each mean is represented by five observations (n = 5) ± SE*.

† *Inoculum used 10^8^ cfu/mL of S. Enteritidis*.

• *Data expressed in Log_10_ cfu/mL*.

### Prophylactic Effects of DFM

#### Effect on *S*. Enteritidis CT and Crop Colonization

The prophylactic effect of DFM (10^4^ cfu/g) on *S*. Enteritidis CT and crop colonization in broiler chickens is shown in Table 2. Although there were no significant differences, there were tendencies in reducing *S*. Enteritidis count, and its incidence in both trials and tissues of chickens in the DFM group as compared to the control group (Table 2). In trial 1, *S*. Enteritidis incidence was reduced by 17% in both CT and crop in DFM group as compared to the control. Similarly, in trial 2, *S*. Enteritidis recovery was decreased by 17 and 23%, respectively, in CT and crop in the DFM group in comparison with the control group. In addition, *S*. Enteritidis count was reduced by less than half log_10_ and more than 1 log_10_ in CT and crop, respectively, in both trials when comparing the DFM group with control group (Table 2).

**Table 2:** Effect of prophylactic administration of DFM (10^4^ cfu/g) on *S*. Enteritidis cecal tonsil (CT) and crop colonization in broiler chickens.

**Treatments**	**CT Log_**10**_ cfu/g**	**CT ± (%)**	**Crop Log_**10**_ cfu/g**	**Crop ± (%)**
**Trial 1**				
Control	4.01 ± 0.29	12/12 (100%)	2.68 ± 0.47	9/12 (75%)
DFM	3.72 ± 0.55	10/12 (83%)	2.11 ± 0.66	6/12 (58%)
**Trial 2**				
Control	3.94 ± 0.22	12/12 (100%)	2.69 ± 0.48	9/12 (75%)
DFM	3.75 ± 0.56	10/12 (83%)	2.08 ± 0.64	5/12 (42%)

*Data expressed in Log_10_ cfu/g (Mean ± SE) of tissue from 12 chickens*.

*Chickens were orally gavaged with 10^7^ cfu of S. Enteritidis per chicken at 6 days old, samples were collected 24 h later*.

*Data expressed as positive/total chickens (%)*.

#### Superoxide Dismutase (SOD) Activity, Serum FITC-d Concentration, and Total Intestinal IgA Levels

The SOD activity, serum FITC-d concentration and total intestinal IgA levels in broiler chickens with or without receiving DFM into the diet are shown in Table 3. DFM significantly reduced SOD activity and total intestinal IgA levels as compared to the control group (*p* < 0.05). However, no significant difference was observed with FITC-d between two groups as shown in Table 3.

**Table 3:** Evaluation of Superoxide dismutase (SOD) activity, serum fluorescein isothiocyanate-dextran (FITC-d) concentration, and total intestinal IgA in broilers chickens that were fed with or without DFM in the diet.

**Treatments**	**SOD (U/mL)**	**FITC-d (μg/mL)**	**IgA (μg/mL)**
Control	4.50 ± 0.31	0.591 ± 0.055	14.21 ± 0.83
DFM	1.97 ± 1.85	0.664 ± 0.063	10.57 ± 0.82

*Samples were collected 24 h post-S. Enteritidis challenge*.

*Data expressed Mean ± SE from 12 chickens, where different letters indicate statistical significant difference at p < 0.05*.

### Therapeutic Effects of DFM

#### Effect on *S*. Enteritidis CT and Crop Colonization

The therapeutic effect of DFM (10^4^ cfu/g) on *S*. Enteritidis CT and crop colonization in broiler chickens is shown in Table 4. Although there were no significant differences, there were tendencies in reducing *S*. Enteritidis count and its incidence in both ages and tissues of chickens in DFM group as compared to the control group (Table 4). At 3-day old, the *S*. Enteritidis count and its incidence in CT were reduced by ~2 log_10_ and 25%, respectively, by DFM group as compared to the control group. In addition, at 10-d old, DFM reduced the *S*. Enteritidis count in CT and crop by more than 1 log_10_ as compared to the control group, while the incidence of *S*. Enteritidis was decreased by 17 and 16%, respectively (Table 4).

**Table 4:** Effect of therapeutic administration of DFM (10^4^ cfu/g) on *S*. Enteritidis cecal tonsil (CT) and crop colonization in broiler chickens.

**Treatments**	**CT Log_**10**_ cfu/g**	**CT ± (%)**	**Crop Log_**10**_ cfu/g**	**Crop ± (%)**
**Trial 3-d**				
Control	6.44 ± 0.15	12/12 (100%)	3.18 ± 0.46	10/12 (83%)
DFM	4.66 ± 0.82	9/12 (75%)	3.05 ± 0.45	10/12 (83%)
**Trial 10-d**				
Control	6.61 ± 0.21	12/12 (100%)	2.93 ± 0.65	7/12 (58%)
DFM	5.49 ± 0.76	10/12 (83%)	1.78 ± 0.65	5/12 (42%)

*Data expressed in Log_10_ cfu/g (Mean ± SE) of tissue from 12 chickens*.

*Chickens were orally gavaged with 10^4^ cfu of S. Enteritidis per chicken at 1 day old; samples were collected 3 and 10 days later*.

*Data expressed as positive/total chickens (%)*.

#### SOD Activity, Serum FITC-d Concentration, and Total Intestinal IgA Levels

The SOD activity, serum FITC-d concentration and total intestinal IgA levels in broiler chickens with or without receiving DFM into the diet at day 10 post-*S*. Enteritidis challenge are shown in Table 5. DFM significantly reduced FITC-d and intestinal IgA levels as compared to the control (*p* < 0.05). In the case of SOD activity, there was a numerical reduction in the DFM group compared to the control group; however, no significant difference was observed.

**Table 5:** Evaluation of Superoxide dismutase (SOD) activity, serum fluorescein isothiocyanate-dextran (FITC-d) concentration, and total intestinal IgA in broilers chickens with or without receiving DFM into the diet at day 10 post-*S*. Enteritidis challenge.

**Treatments**	**SOD (U/mL)**	**FITC-d (μg/mL)**	**IgA (μg/mL)**
Control	10.34 ± 0.67	0.700 ± 0.020	14.34 ± 2.81
DFM	9.29 ± 0.88	0.531 ± 0.013	6.21 ± 2.31

*Data expressed as Mean ± SE from 12 chickens, where different letters indicate statistical significant difference at p < 0.05*.

#### Cecal Microbiota

Summarization of the OTU table resulted a total of 441,934 reads that range from 27,654 to 43,856 reads per sample. The total number of OTUs after data filtering was 1,108.

##### Taxonomic Assignments

**Figure 1:**
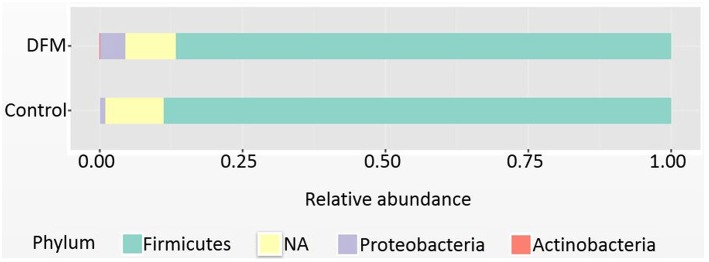
Relative abundance of major phyla recovered in ceca of broiler chickens at day 10 from two different treatment groups (control and DFM). NA refers to those reads that could not be assigned to any phyla.

*Phylum level Firmicutes* were found as a predominant phylum in both groups (Control group, 88.71%; DFM group, 86.68%) followed by *Proteobacteria* and *Actinobacteria* as shown in Figure 1. *Actinobacteria* were significantly reduced in the DFM group as compared to the control group (*p* < 0.05).

**Figure 2:**
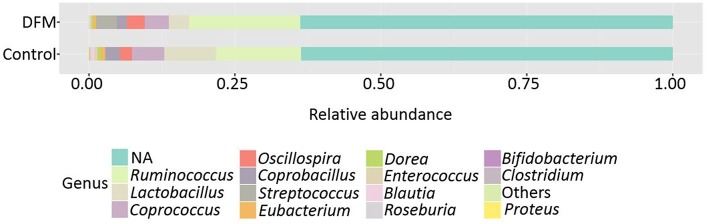
Relative abundance of major genera recovered in ceca of broiler chickens at day 10 from two different treatment groups (control and DFM). NA refers to those reads that could not be assigned to any genera. Genera having counts <100 are merged together in “Others”.

*Genus level* The relative abundance of different genera present in the control and DFM groups is shown in Figure 2. *Ruminococcus* was found as a predominant genus in both groups (Control group, 14.48%; DFM group, 19.14%), followed by *Lactobacillus* (Control group, 8.91%; DFM group, 3.40%), and *Streptococcus* (Control group, 0.15%; DFM group, 3.68%) in control and DFM, respectively.

The genera *Bifidobacterium* (Control group, 0.094%; DFM group, not detected), *Roseburia* (Control group, 0.19%; DFM group, 0.035%), *Proteus* (Control group, 0.07%; DFM group, not detected), and cc_115 (Control group, 0.04%; DFM group, not detected) were significantly decreased, while the genus *Streptococcus* was significantly enriched in the DFM group as compared to the control group (MetagenomeSeq, *p* < 0.05). In addition, some of the notable genera such as *Enterococcus, Dorea, Coprobacillus, Coprococcus, Eubacterium*, and *Blautia* were numerically reduced in the DFM group as compared to the control group.

##### Microbial Diversities analysis

**Figure 3:**
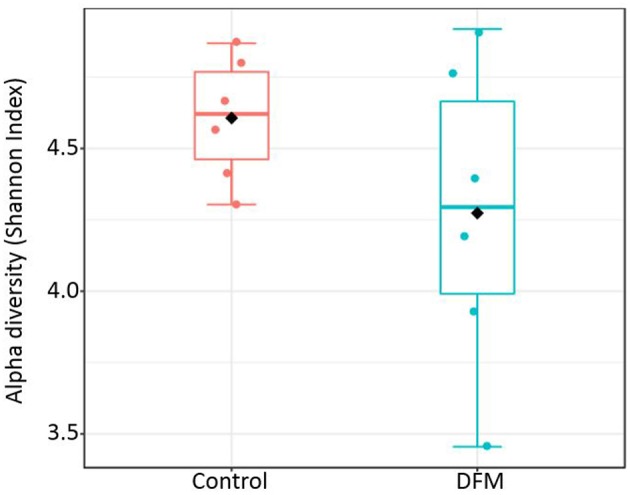
Alpha diversity of two different groups (control and DFM) as measured by Shannon Index. No significant difference was observed between them (*T*-test, *p* > 0.05). The diamond shape represents the mean value in each group.

*Alpha diversity* Alpha diversity of control and DFM groups as measured by Shannon index is shown in Figure 3. The average Shannon index in the control group was 4.61 ± 0.09 (Mean ± SE) and 4.27 ± 0.22 in the case of the DFM group. However, there was no significant difference observed between both groups.

**Figure 4:**
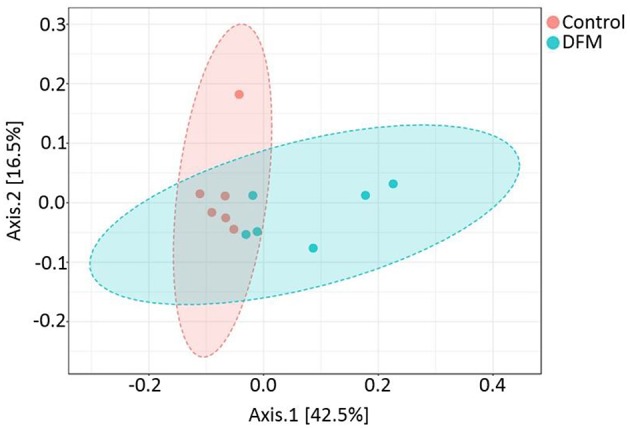
PCoA plot showing difference in microbial community structure between control and DFM groups (ANOSIM; R = 0.35 and *p* < 0.01).

*Beta diversity* Beta diversity between control and DFM groups as measured by weighted UniFrac metric is illustrated in PCoA plot (Figure 4). Analysis of similarities (ANOSIM) showed significant difference in microbial community structure between the two groups (R = 0.35, *p* < 0.01).

**Figure 5:**
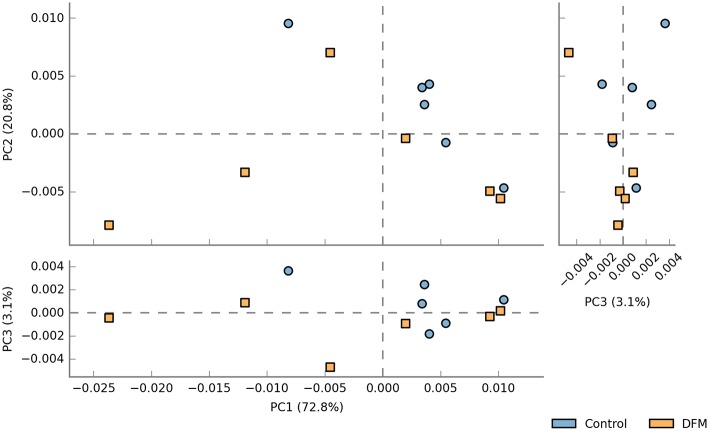
PCA plot comparing third level KEGG pathways between control and DFM groups. The third level KEGG pathways were predicted using PICRUSt followed by the generation of PCA plot using STAMP.

**Figure 6:**
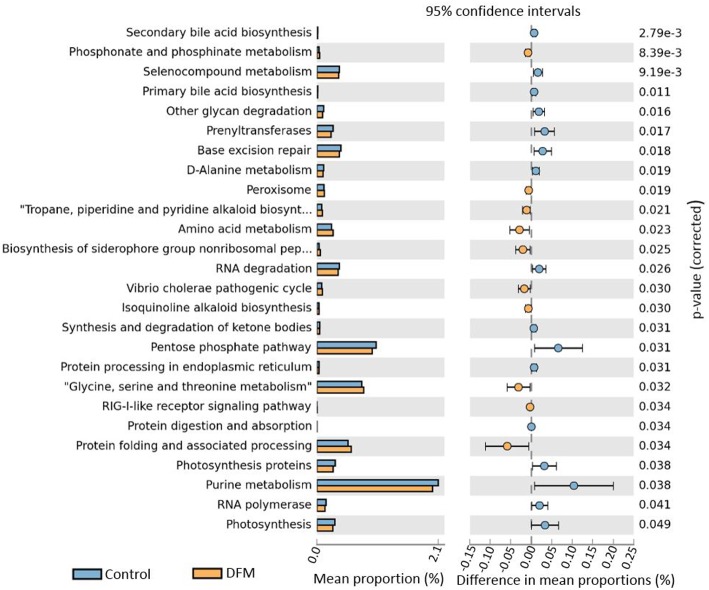
Extended error bar plot generated by STAMP showing differential abundant third level KEGG pathways between control and DFM group. Only significant features with *p* < 0.05 (Welch's *t*-test) were included in the plot.

*Functional potentialities of cecal bacterial community* The predicted functions of cecal microbiota in the control and DFM groups by PICRUSt and their analysis by STAMP are shown in Figures 5, 6. The PCA plot shows that the third level KEGG pathways of the DFM group are relatively distinct in comparison to the control group (Figure 5). More specifically, many bacterial genes that are involved in various metabolic pathways such as bile acid synthesis (primary and secondary), carbohydrate metabolism (pentose phosphate pathway and other glycan degradation), and nucleotide metabolism (purine) were predicted to be enriched in the control group. On the other hand, bacterial genes that could involve in amino acid metabolism (Glycine, Serine, and Threonine) and alkaloid biosynthesis (isoquinoline, tropane, piperidine, and pyridine alkaloids) were predicted to be enriched in the DFM group (Figure 6).

## Discussion

Previous research reported nontyphoidal *Salmonella* sps., *Clostridium perfringens, Campylobacter* sps., and *Escherichia coli* as some of the most important foodborne bacterial pathogens in the United States (49). The overall health-related costs associated with foodborne illness from those pathogens was estimated to be around $51.0 and $77.7 billion based on the basic and enhanced model, respectively, as described earlier (50). Nontyphoidal *Salmonella* sp. was reported as a major causative agent for hospitalization and deaths of patients in the United States (49). *S*. *Enterica* serotype Enteritidis (*S*. Enteritidis) which emerged as an important human illness during 1980s is currently one of the most common nontyphoidal *Salmonella* serotypes worldwide, especially in developed countries (51). Poultry and their products (eggs and meat) are considered as one of the most important sources of *S*. Enteritidis infection in humans; however, *S*. Enteritidis was also isolated from non-poultry sources such as market hog carcass, steer and heifer carcass, cow and bull carcass, and ground beef (52–54).

Several studies have been conducted with the objective to reduce *S*. Enteritidis load in poultry and their products using various approaches such as antibodies, bacteriophages, probiotics, prebiotics, vaccines, and integrated farm management (55–59). In the present study, we evaluated the effects of Norum™ (DFM) to reduce *S*. Enteritidis colonization using both *in vitro* and *in vivo* trials in broiler chickens. Our previous study using an *in vitro* digestion model showed a reduction of *C. perfringens* by the isolates used in Norum™ in different non-corn based diets demonstrating their antibacterial property against this Gram-positive bacteria (24). The antimicrobial activity of various species of *Bacillus*, including *B*. *subtilis* and *B*. *amyloliquefaciens*, were studied elsewhere and found to be effective mainly against Gram-positive bacteria (60–63). In the current study, we also observed the reduction of *S*. Enteritidis by DFM in the intestinal compartment simulated in the model and in both proventriculus and intestinal compartments, when using 10^4^ spores/g and 10^6^ spores/g DFM, respectively. The *Salmonella* Enteritidis colonization was reduced by more than 7 log_10_ cfu/mL and brought to an undetectable level in the intestinal compartment when DFM was used at 10^6^ spores/g of feed, suggesting its more noticeable antibacterial effects at a higher dose. These findings further suggest that DFM exhibits a wide range of antibacterial activities which can be effective for both Gram-positive and negative bacteria. Although the detailed mechanism is not well-understood, these antibacterial properties of DFM might be achieved not only through competitive exclusion and production of antimicrobial peptides (AMPs), but also might be indirectly through one or several beneficial effects exhibited by them including secretion of exogenous enzymes, alternation of immunity, gut microbiota, and morphology (23, 24, 64, 65). The AMPs secreted by *Bacillus* sps. are diverse in nature with different chemical structure (60) and include bacteriocins, glycopeptides, lipopeptides, and cyclic peptides (61).

The antibacterial activity of *Bacillus* isolates in Norum™ against *Clostridium perfringens* (24), *S*. Enteritidis, *Escherichia coli*, and *Clostridium difficile* (64) was evaluated earlier using an *in vitro* model and reported as promising DFM candidates. In addition, dietary supplementation with DFM (10^6^ spores/g) mitigated the negative impacts of necrotic enteritis in broiler chickens using a laboratory challenge model (35). Therefore, considering that the model of necrotic enteritis is more severe than a *Salmonella* infection model and *in vitro* results, in this study, we evaluated the therapeutic and prophylactic effects of those isolates in Norum™ (10^4^ spores/g) against *S*. Enteritidis CT and crop colonization in broiler chickens. Although there were no significant differences, there were tendencies in reducing *S*. Enteritidis count and its incidence in both ages (3 and 10 days) and tissues (CT and crop) of chickens by DFM as compared to the control during the therapeutic study. Similar tendencies were also reported in both trials during the prophylactic study. This may be due to the lower dose of *Bacillus* spores (10^4^ spores/g of feed) used during the *in vivo* trials, because the antibacterial effect was more pronounced with higher dose compared to the lower dose as demonstrated by the *in vitro* digestion model (Table 1). A similar antimicrobial dose-dependent response of *Bacillus*-DFM against necrotic enteritis was observed earlier, where the higher dose (10^6^ cfu/g of feed) mitigated negative impacts of NE more than the lower dose (10^4^ cfu/g of feed) (66).

Several enteric pathogens including *Salmonella* sps. disrupt the intestinal tight junctions leading to the increase in gut permeability; commonly known as “leaky gut” (67, 68). Serum FITC-d increases with inflammation and is considered as a good indicator to measure enteric inflammation induced gut permeability in broiler chickens (69). The significant reduction (*p* < 0.05) of serum FITC-d level by DFM as compared to the control group in the therapeutic study might be due to the alleviation of negative impacts of *S*. Enteritidis by increasing the regulation of tight junction proteins (23, 70). Antioxidant enzymes such as SOD play a vital role to degrade superoxide anions and hydrogen peroxide produced during an inflammatory process. There was a significant (*p* < 0.05) and numerical increase of SOD activity in the control group of the prophylactic and therapeutic study, respectively, when compared to the group treated with DFM. The increased SOD activity in the control group could be related to the response to increased oxidative stress due to severe intestinal damage caused by *S*. Enteritidis, since SOD plays a key role in the reduction of oxidative stress (71). Similarly, the significant increase in IgA level (*p* < 0.05) in both *in vivo* trials might be associated with disruption of intestinal epithelium, since secretion of intestinal IgA serves as the first line of defense to protect the intestinal epithelium from enteric toxins and pathogenic microorganism, as well as to antagonize the inflammatory processes and enhance the non-specific defense mechanisms (32, 72). In contrast, the decrease of SOD activity and IgA level by DFM could be related to its anti-inflammatory and immune modulating properties to mitigate the negative impacts of *S*. Enteritidis, reducing the gut morphological and immunological alterations through expression of the cytoprotective proteins and modulation of various cytokines (19, 23, 73–76).

Along with the advancement in sequencing technologies, the cost of sequencing has significantly reduced recently, making microbiota studies more affordable. It is now a well-accepted fact that the gut microbiota plays a key role in health and diseases of both humans and animals, which has been reviewed elsewhere (77–80). Although detailed mechanisms are unknown, the supplementation of various alternatives to antibiotics including *Bacillus*-DFM can improve overall intestinal health and growth in chickens (24, 35), probably due to the modulation of the gut microbiota, which is one of the important mechanisms of action exhibited by alternatives to antibiotics in order to exert beneficial effects on the host (2, 23, 81–83). Moreover, the inclusion of *Bacillus*-DFM has been shown to alter the cecal (20) and ileal (21) microbiota in broiler chickens.

The cecum of the chicken harbors the greatest bacterial diversity and is an important organ for water regulation and production of short chain fatty acids (SCFA) through carbohydrate fermentation (23, 84). The ceca of young chickens are mainly dominated by the phylum *Firmicutes, Proteobacteria*, and *Actinobacteria*, whereas the relative abundance of *Bacteriodetes* increases with age and was detected only after 15 days in broiler chickens (85). We also reported *Firmicutes* as the dominant phyla in both groups followed by *Proteobacteria* and *Actinobacteria*. *Actinobacteria* were significantly lowered by the DFM, which could be due to the antibacterial activity of DFM against *S*. Enteritidis since *Actinobacteria* were increased in chickens infected with *S*. Enteritidis (5, 86). The genus *Proteus* and the genus cc_115 of the family Erysipelotrichaceae were significantly higher in the control group as compared to the DFM. The increased abundance of *Proteus* and cc_115 was associated with necrotic enteritis in broiler chickens (87). Similarly, the genus *Proteus* and the bacterial family Erysipelotrichaceae were found to be associated with intestinal dysbiosis in humans as reported in the Disbiome^R^ database (88). Thus, the increase of *Proteus* and cc_115 in the control might be associated with gut dysbiosis and inflammation caused by *S*. Enteritidis (89), whereas their decrease in the DFM group might be due to the antibacterial property of DFM. Furthermore, the increase of *Bifidobacterium* and *Roseburia* in the control group might be due to the inflammatory response, since these genera were found to have anti-inflammatory properties (90, 91). A significant increase in *Bifidobacterium* after *S*. Enteritidis inoculation was also reported earlier in chickens (92). Although some of the species of *Streptococcus* cause infection in poultry (93, 94) they are commensal organisms present in the GI tract of chickens and have been used as potential probiotics (95, 96) because of their ability to reduce pathogen colonization through competitive exclusion and reduction of the pH through lactic acid production (97). Thus, increase in *Streptococcus* by DFM in the present study may be playing a vital role in reducing the colonization and incidence of *S*. Enteritidis; however, a higher resolution to the strain level is needed to understand the actual effects as two strains of the same species can carry out completely opposite roles (98).

DFM not only affected the bacterial composition in the ceca of broiler chickens but also the community structure as indicated by the beta diversity analysis. However, in the case of alpha diversity, although there was numerically higher diversity in the control group, no significant difference was observed between the two groups. This may be related to one of the theories that the DFM promotes growth of the host by reducing the number and diversity of the commensal microbiota, which will allow increased nutrient utilization by intestinal epithelial cells and lower detrimental effects of microbial metabolites (99). These regulations by DFM might be achieved through changes in bacterial genes involved in various metabolic pathways (Figures 5, 6). One of the important metabolic pathways predicted to be enriched in the control group was bile acid synthesis. Bile acids are considered as important regulators of the gut microbiota and reduced levels of bile acids in the gut are associated with bacterial overgrowth and intestinal inflammation (100, 101). Enrichment of the bile acid synthesis pathway in the control group might be a response to the lower level of bile acids and inflammation caused by *S*. Enteritidis and other dysbiosis associated bacteria colonization in the gut. Similarly, another glycan degradation pathway was enriched in the control group, and this might be related to the response of mucinogeneis as a result of *S*. Enteritidis inflammation and the overgrowth of *Bifidobacterium* in the control group, which can degrade the host-derived glycans (102). Amino acids serve as precursors for microbial-derived SCFA such as acetate, propionate, and butyrate, which has been reviewed elsewhere (103). Meanwhile, the increase in metabolic pathways associated with the metabolism of amino acids (glycine, serine, and threonine) in the DFM group could be related to the amino acid fermenting ability of the *Bacillus*-DFM (104) to produce SCFA. SCFA serves as nutrients for colonocytes and other gut epithelial cells and plays a key role in shaping the gut microbiota of the host (105). Future investigation of the effects of DFM in the *Salmonella* challenged model by metagenomics and metabolomics analysis will reveal more functional potentialities of DFM.

In summary, the overall results of the present study suggest that the *Bacillus*-DFM (Norum™) can be used for the prevention and treatment of *S*. Enteritidis infection since it has the potential to reduce *S*. Enteritidis colonization and mitigate its negative effects in broiler chickens. These effects of Norum™ could be achieved through mechanism(s) that might involve the modulation of gut microbiota and their metabolic pathways. The effects of Norum™ against *S*. Enteritidis at a higher dose (10^6^ spores/g) may disclose more promising results and are currently under evaluation.

## Data Availability

The raw data supporting the conclusions of this manuscript will be made available by the authors, without undue reservation, to any qualified researcher.

## Ethics Statement

All animal handling procedures complied with the Institutional Animal Care and Use Committee (IACUC) at the University of Arkansas, Fayetteville (protocol #18030).

## Author Contributions

BA and GT-I designed the experiments and wrote the first version of the manuscript. BA, DH-P, and BS-C performed the experiment. YK, MA, JL, and BH aided in the analysis and interpretation of the data and supervised the project. GT-I, BA, JL, and XH-V contributed to editing the final version of the manuscript. All the authors reviewed and finally approved the manuscript.

### Conflict of Interest Statement

MA was employed by Eco-Bio LLC. The remaining authors declare that the research was conducted in the absence of any commercial or financial relationships that could be construed as a potential conflict of interest.
