# Supplementary material for: Evaluation of the Antimicrobial and Anti-inflammatory Properties of Bacillus-DFM (Norum™) in Broiler Chickens Infected With Salmonella Enteritidis
Source: Front Vet Sci. 2019 Aug 27;6:282. doi: 10.3389/fvets.2019.00282 (PMC6718558; doi:10.3389/fvets.2019.00282)
Supplement: Supplementary file 1 [file Table_1.DOCX]

**Supplementary Table 1.** Ingredient composition and nutrient content of a basal starter diet used in the experiments on as-is basis.

| **Item** | | **Corn soybean-based diet** |
| --- | --- | --- |
| Ingredients (g/kg) |  | |
| Corn | 574.5 | |
| Soybean meal | 346.6 | |
| Poultry oil | 34.5 | |
| Dicalcium phosphate | 18.6 | |
| Calcium carbonate | 9.9 | |
| Salt | 3.8 | |
| DL-Methionine | 3.3 | |
| L-Lysine HCL | 3.1 | |
| Threonine | 1.2 | |
| Choline chloride 60 % | 2.0 | |
| Vitamin premix^1^ | 1.0 | |
| Mineral premix^2^ | 1.0 | |
| Antioxidant^3^ | 0.5 | |
| **Calculated analysis** |  | |
| Metabolizable energy (MJ/kg) | 12.7 | |
| Crude protein (g/kg) | 221.5 | |

^1^Vitamin premix supplied per kg of diet: Retinol, 6 mg; cholecalciferol, 150 µg; dl-α-tocopherol, 67.5 mg; menadione, 9 mg; thiamine, 3 mg; riboflavin, 12 mg; pantothenic acid, 18 mg; niacin, 60 mg; pyridoxine, 5 mg; folic acid, 2 mg; biotin, 0.3 mg; cyanocobalamin, 0.4 mg.

^2^Mineral premix supplied per kg of diet: Mn, 120 mg; Zn, 100 mg; Fe, 120 mg; copper, 10 to 15 mg; iodine, 0.7 mg; selenium, 0.2 mg; and cobalt, 0.2 mg.

^3^Ethoxyquin.
